# Prognostic model development and molecular subtypes identification in bladder urothelial cancer by oxidative stress signatures

**DOI:** 10.18632/aging.205499

**Published:** 2024-02-01

**Authors:** Ying Dong, Xiaoqing Wu, Chaojie Xu, Yasir Hameed, Mostafa A. Abdel-Maksoud, Taghreed N. Almanaa, Mohamed H. Kotob, Wahidah H. Al-Qahtani, Ayman M. Mahmoud, William C. Cho, Chen Li

**Affiliations:** 1 Department of Urology, The First Affiliated Hospital of Shenzhen University, Shenzhen Second People’s Hospital, Shenzhen, China; 2 Department of Oncology, Guang’anmen Hospital, China Academy of Chinese Medical Sciences, Beijing, China; 3 Department of Urology, Peking University First Hospital, Peking University, Beijing, China; 4 Department of Biochemistry, Biotechnology, The Islamia University of Bahawalpur, Pakistan; 5 Botany and Microbiology Department, College of Science, King Saud University, Riyadh, Saudi Arabia; 6 Department of Pharmaceutical Sciences, Division of Pharmacology and Toxicology, University of Vienna, Vienna, Austria; 7 Department of Food Sciences and Nutrition, College of Food and Agricultural Sciences, King Saud University, Riyadh, Saudi Arabia; 8 Department of Life Sciences, Faculty of Science and Engineering, Manchester Metropolitan University, Manchester, United Kingdom; 9 Department of Clinical Oncology, Queen Elizabeth Hospital, Kowloon, Hong Kong, China; 10 Department of Biology, Chemistry, Pharmacy, Free University of Berlin, Berlin, Germany

**Keywords:** oxidative stress, bladder urothelial cancer, tumor microenvironment, immunotherapy

## Abstract

Background: Mounting studies indicate that oxidative stress (OS) significantly contributes to tumor progression. Our study focused on bladder urothelial cancer (BLCA), an escalating malignancy worldwide that is growing rapidly. Our objective was to verify the predictive precision of genes associated with overall survival (OS) by constructing a model that forecasts outcomes for bladder cancer and evaluates the prognostic importance of these genetic markers.

Methods: Transcriptomic data were obtained from TCGA-BLCA and GSE31684, which are components of the Cancer Genome Atlas (TCGA) and Gene Expression Omnibus (GEO), respectively. To delineate distinct molecular subtypes, we employed the non-negative matrix factorization (NMF)method. The significance of OS-associated genes in predicting outcomes was assessed using lasso regression, multivariate Cox analysis, and univariate Cox regression analysis. For external validation, we employed the GSE31684 dataset. CIBERSORT was utilized to examine the tumor immune microenvironment (TIME). A nomogram was created and verified using calibration and receiver operating characteristic (ROC) curves, which are based on risk signatures. We examined variations in clinical characteristics and tumor mutational burden (TMB) among groups classified as high-risk and low-risk. To evaluate the potential of immunotherapy, the immune phenomenon score (IPS) was computed based on the risk score. In the end, the pRRophetic algorithm was employed to forecast the IC50 values of chemotherapy medications.

Results: In our research, we examined the expression of 275 genes associated with OS in 19 healthy and 414 cancerous tissues of the bladder obtained from the TCGA database. As a result, a new risk signature was created that includes 4 genes associated with OS (RBPMS, CRYAB, P4HB, and PDGFRA). We found two separate groups, C1 and C2, that showed notable variations in immune cells and stromal score. According to the Kaplan-Meier analysis, patients classified as high-risk experienced a considerably reduced overall survival in comparison to those categorized as low-risk (P<0.001). The predictive capability of the model was indicated by the area under the curve (AUC) of the receiver operating characteristic (ROC) curve surpassing 0.6. Our model showed consistent distribution of samples from both the GEO database and TCGA data. Both the univariate and multivariate Cox regression analyses validated the importance of the risk score in relation to overall survival (P < 0.001). According to our research, patients with a lower risk profile may experience greater advantages from using a CTLA4 inhibitor, whereas patients with a higher risk profile demonstrated a higher level of responsiveness to Paclitaxel and Cisplatin. In addition, methotrexate exhibited a more positive outcome in patients with low risk compared to those with high risk.

Conclusions: Our research introduces a novel model associated with OS gene signature in bladder cancer, which uncovers unique survival results. This model can assist in tailoring personalized treatment approaches and enhancing patient therapeutic effect in the management of bladder cancer.

## INTRODUCTION

Bladder urothelial carcinoma (BLCA), the primary cancer in the urinary system [1], develops from the uroepithelium, which is the cell layer that covers the inner surface of the bladder [2]. In areas such as the United States and Western Europe, this specific type of carcinoma accounts for the majority of instances of bladder cancer, approximately 90% [3]. The local immune response is induced by the administration of Bacillus Calmette-Guerin infused into the bladder, which improves early-stage BLCA [4, 5]. However, this immunotherapy is merely applicable to superficial bladder cancer. Since that time, there has been limited progress in the treatment of bladder cancer, with only a few significant advancements [6]. Administering neoadjuvant chemotherapy before performing radical cystectomy can enhance patient survival following the surgery. Various immune checkpoint inhibitors have been utilized in the treatment of advanced bladder cancer. Moreover, the initial demonstration has shown the safety and effectiveness. Currently, Pembrolizumab, Nivolumab, Atezolizumab, Duvalizumab, and Avelumab have been approved for use as second-line treatment following the ineffectiveness of platinum-based chemotherapy [7].

Metabolic reprogramming enables tumor cells to obtain the necessary substance and energy for rapid proliferation [8]. The reprogramming of tumor metabolism is linked to the generation of reactive oxygen species (ROS) and the stimulation of antioxidant mechanisms that cancer cells need elevated ROS levels for sustaining rapid growth [9]. Oxidative stress (OS) is induced by ROS accumulation and redox status dysfunction. OS refers to a condition where there exists a disparity between the effects of oxidation and antioxidants within the body, leading to oxidation, which in turn causes infiltration of neutrophils, heightened secretion of proteolytic enzymes, and the generation of substantial quantities of oxidative intermediates. The body experiences oxidative stress as a detrimental outcome caused by free radicals, which is acknowledged as a significant factor in the process of aging and the development of diseases. The assessment of oxidative stress can be done by directly measuring ROS or indirectly by evaluating the damage caused to lipids, proteins, and nucleic acids due to excessive production of ROS. While the direct assessment of ROS is the preferred approach, alternative methods are frequently utilized due to the enduring stability of damage indicators on biomolecules in contrast to the fleeting nature of ROS. ROS directly causes oxidative changes to the protein, leading to abnormalities in its structure or function [10]. A mutation of bladder cell DNA is responsible for urothelial bladder carcinoma. The proper level of ROS is involved in the control of various signaling pathways as a secondary messenger, while excessive ROS causes damage to genomic and mitochondrial DNA and disrupts signaling pathways [11, 12]. According to the report, the LRPPRC, which regulates the metabolism of mitochondrion mRNA, has been identified as an autonomous predictor of BLCA and demonstrates resistance to oxidation [13]. The growth and movement of 253J-BV bladder cancer cells, both sensitive and resistant to cisplatin, are hindered by Aila, a natural extract. This is achieved by decreasing the levels of post-translational Nrf2 and YAP proteins as well as the content of superoxide anions [14]. Hence, the oxidation-reduction condition might serve as a potential predictive factor for individuals with bladder carcinoma.

We examined the impact of OS on bladder cancer by analyzing data from the TCGA-BLCA and GSE31684 groups in our research. Integrating clinical features with a risk signature of genes associated with overall survival, our objective was to create a prognostic model and a nomogram. To identify distinct molecular subtypes, we applied the Non-Negative Matrix Factorization (NMF) method. The purpose of this model is to forecast the likelihood of survival after 1, 3, and 5 years for individuals diagnosed with bladder cancer. Furthermore, we investigated the combined impact of the risk score, infiltration of immune cells into tumors, responsiveness to immunotherapy, tumor mutational burden (TMB), and sensitivity to drugs, highlighting the significance of our discoveries in a clinical context.

## MATERIALS AND METHODS

### Data download and extraction

From the TCGA-BLCA database, we acquired the transcriptome profiles of 414 BLCA samples and 19 samples of normal bladder tissue. After excluding bladder cancer cases with no clinical information, we ultimately retrieved transcriptome profiling for 406 samples with complete information. The TCGA database provided clinical annotations including clinicopathological characteristics and survival rate. To validate externally, we employed information from the GSE31684 group, acquired from the GEO repository, comprising microarray data from 93 instances of bladder cancer that were examined using the Affymetrix Human Genome U133 Plus 2.0 Array (Platform GPL570). When collecting OS-related genes from the Genecards database and published literature (Supplementary Table 1), a sum of 275 potential genes was incorporated.

### Selection of differentially expressed genes (DEGs) related to OS

To identify differentially expressed genes (DEGs) associated with oxidative stress (OS), we employed the ‘limma’ R package to analyze the gene expression levels in bladder cancer in comparison to normal bladder tissues. To identify significant DEGs, the criteria included an FDR below 0.05 and an absolute log2 FC exceeding 1. In this context, a log2FC above 1 indicated gene upregulation, whereas a log2FC below -1 indicated gene downregulation in tumor samples.

### Molecular subtype identification using non-negative matrix factorization (NMF) algorithm

To perform molecular subtype identification, we employed the NMF algorithm using the ‘NMF’ R package. The decision to use NMF was motivated by its ability to identify and group the inherent characteristics present in tumor samples, which is crucial for precise molecular subtyping. Additionally, this method aided in the retrieval of biologically significant correlation coefficients, offering a more profound understanding of the fundamental biology of tumors. To ensure the reliability and accuracy of the subtype classification, the selection of the best k value, which ranged from 2 to 10, was guided by finding a balance between stability and clustering performance. This involved evaluating the covariance and the sum of squared residuals (RSS).

### Definition and comparison of tumor-infiltrating immune cells

In BLCA samples, we employed the MCP-counter to examine immune cells that have infiltrated the tumor microenvironment. The process included normalizing gene expression data, utilizing an MCP-counter to measure immune and stromal cells, and comparing these scores to detect important tumor-infiltrating cells.

### Construction and validation of OS-related genes prognosis features

To identify DEGs with potential prognostic significance, we utilized Cox regression analyses in R with the assistance of the ‘survival’ and ‘survminer’ packages [15]. By utilizing this method, we were able to identify possible genes that could be examined in more detail. Afterward, we utilized the Lasso technique, employing the ‘glmnet’ software in R, to build a predictive model. This model integrated these candidate genes, focusing on their potential predictive power in relation to patient outcomes [16]. The risk score was calculated utilizing the equation [17].


$$Risk\;score=\sum_{i=1}^{n}\left(coef\text{​}i*Expi\right)$$

In our study, we utilized a particular mathematical formula to determine the risk score. This formula involves multiplying the expression levels of each chosen candidate gene by their respective regression coefficients obtained from the multivariate Cox regression analysis. Adding up these products provides the risk score for every individual. Patients were categorized into high-risk and low-risk groups based on these scores, using the median risk score as the dividing factor.

To validate our prognostic model, we trained it using data from TCGA and then tested it on data from the GEO database. Confirmation of the model’s stability and effectiveness was achieved by analyzing the Receiver Operating Characteristic (ROC) curve. In order to establish the risk score as a crucial element in forecasting prognosis, we conducted both univariate and multivariate Cox regression analyses. Furthermore, the R package ‘heatmap’ was employed to visually depict and examine the correlation between the risk score and different clinical factors.

### Gene set enrichment analysis (GSEA)

In order to explore the functional annotations linked to high-risk and low-risk groups in our investigation, we employed the Gene Set Enrichment Analysis (GSEA) program, specifically version 4.1. The examination was performed utilizing the c2.cp.kegg.v7.4.symbols gene set compilation [18]. Results were deemed statistically significant if the p-value was below 0.05. To ensure effective visualization and interpretation, we have chosen and presented the top eight significant results obtained from this analysis.

### Establishment and verification of the nomogram

The nomogram was developed using the ‘rms’ package in R, and its visualization was performed with the ‘regplot’ package [19]. The model integrated the risk scores from the nomogram and the clinical characteristics of the patients. The nomogram offers a numerical instrument to forecast the overall lifespan within 1, 3, and 5-year timeframes. Calibration curves were created and examined to evaluate the precision and effectiveness of the nomogram. Verifying the effectiveness and applicability of the nomogram in clinical prognosis was of utmost importance in this process.

### Correlation between TMB and risk scores

Somatic mutation data was obtained from the TCGA-BLCA cohort and then assessed using the R program ‘maftools’ [20] for somatic non-synonymous point mutations. TMB refers to the aggregate count of genetic coding mistakes, base substitutions, gene insertions, or deletions identified per million base pairs.

### Correlation of risk score with tumor immune infiltration microenvironment (TIME) characterization

To evaluate the presence of immune cells in the tumor microenvironment [21], we employed a total of 7 techniques: XCELL, TIMER, QUANTISEQ, MCP-counter, EPIC, CIBERSORT, and CIBERSORT-ABS. To visualize the aforementioned results, the package ‘ggpubr’ was utilized. Every one of these techniques provides a distinct strategy for evaluating the presence of immune cells within the tumor microenvironment. XCELL and TIMER offer information on the prevalence and positioning of immune cells, QUANTISEQ and MCP-counter concentrate on measuring immune cell populations, EPIC expands these evaluations to encompass a wider array of cell types, whereas CIBERSORT and CIBERSORT-ABS employ gene expression profiles to deduce the makeup of cell mixtures in tumor samples. By adopting this all-encompassing method, a meticulous assessment of TIME is guaranteed, thereby augmenting comprehension of its correlation with the risk score in BLCA.

### Gene set variation analysis (GSVA)

We obtained two gene sets, the c7.immunesigdb HALLMARK and c2.cp.kegg.v7.4, from the Molecular Signatures Database for our analysis. Furthermore, we have incorporated a compilation of 47 genes renowned for their correlation with immune checkpoint blockade. By utilizing these sets of genes, we conducted GSVA in a standardized manner for every sample in order to measure the comparative level of activity in different pathways, with particular emphasis on immunological characteristics and immune regulatory points. This analysis allowed us to ascertain the functional relevance of these pathways and checkpoints in the context of bladder cancer.

### Anticancer drug sensitivity prediction

In clinical practice, acknowledging chemotherapy and immunotherapy as the main treatment approaches for bladder cancer, our objective was to compare the half-maximal inhibitory concentration (IC50) of medications on BLCA samples between groups categorized as high-risk and low-risk. We used the “pRRophetic” package, which predicts drug sensitivity based on gene expression profiles from TCGA and cell line expression profiles from Genomics of Cancer Drug Sensitivity. By utilizing this method, we were able to evaluate the possible effectiveness of these therapies in various categories of risk, which aids in making personalized decisions regarding treatment. Immune checkpoint inhibitors are a form of therapy used to treat advanced bladder cancer. The Immunopheno Score (IPS) was utilized to evaluate the immunogenicity of tumors in BLCA populations categorized as high-risk and low-risk. The Immune Profiling Score (IPS) was computed using genes linked to immune checkpoint inhibition, utilizing gene information obtained from the Genecards repository.

### Statistical analysis

In our study, we utilized the Kruskal-Wallis test to compare more than two groups and the Wilcoxon test to compare two groups. To assess differences in survival curves, we utilized the Kaplan-Meier log-rank test, a highly effective method for evaluating survival data. The chi-square analysis played a crucial role in investigating the connections between risk group categorizations and the burden of somatic mutations. Furthermore, we employed Spearman’s rank correlation analysis to compute correlation coefficients, offering valuable insights into the associations among different study variables. In order to conduct a more in-depth analysis of the data, specifically regarding the makeup of immune cells, the CIBERSORT algorithm was utilized, with outcomes deemed statistically significant if the p-value was below 0.05. R software (version 4.1.1) was utilized for all statistical analyses, incorporating specific R packages like ‘survival’ for Kaplan-Meier analysis and ‘ggplot2’ for data visualization [22]. A significance level of 0.05 was used to determine statistical significance based on two-sided p-values.

### Data availability

All data used in the study were from the publicly available The Cancer Genome Atlas (TCGA) (https://portal.gdc.cancer.gov/). Informed consent forms are not required for patient data extracted from public databases.

## RESULTS

### Identification of DEGs between normal bladder and BLCA tissues

**Figure 1:**
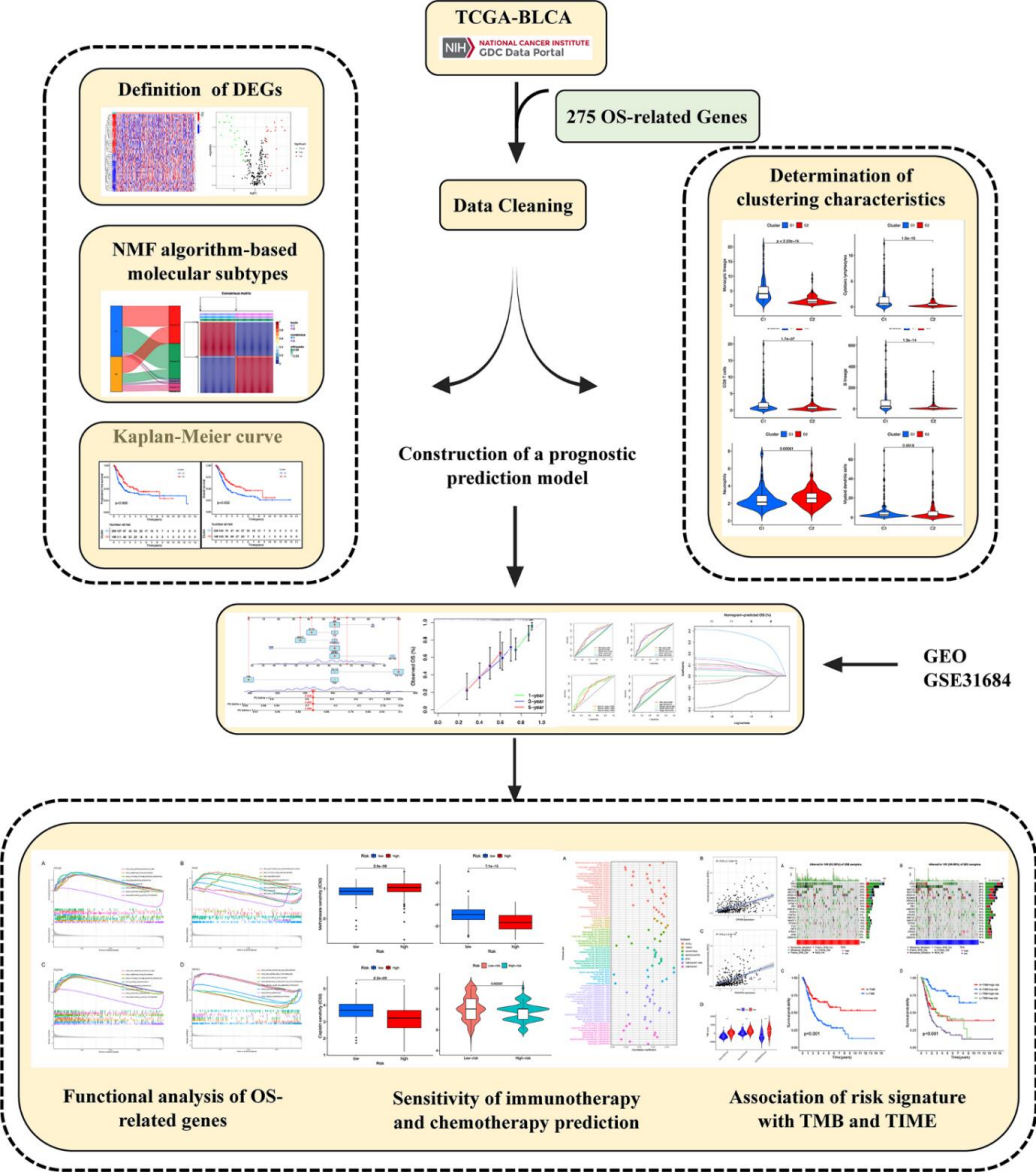
Graphical abstract of construction of a prognostic index associated with oxidative stress signatures in bladder urothelial cancer.

**Figure 2:**
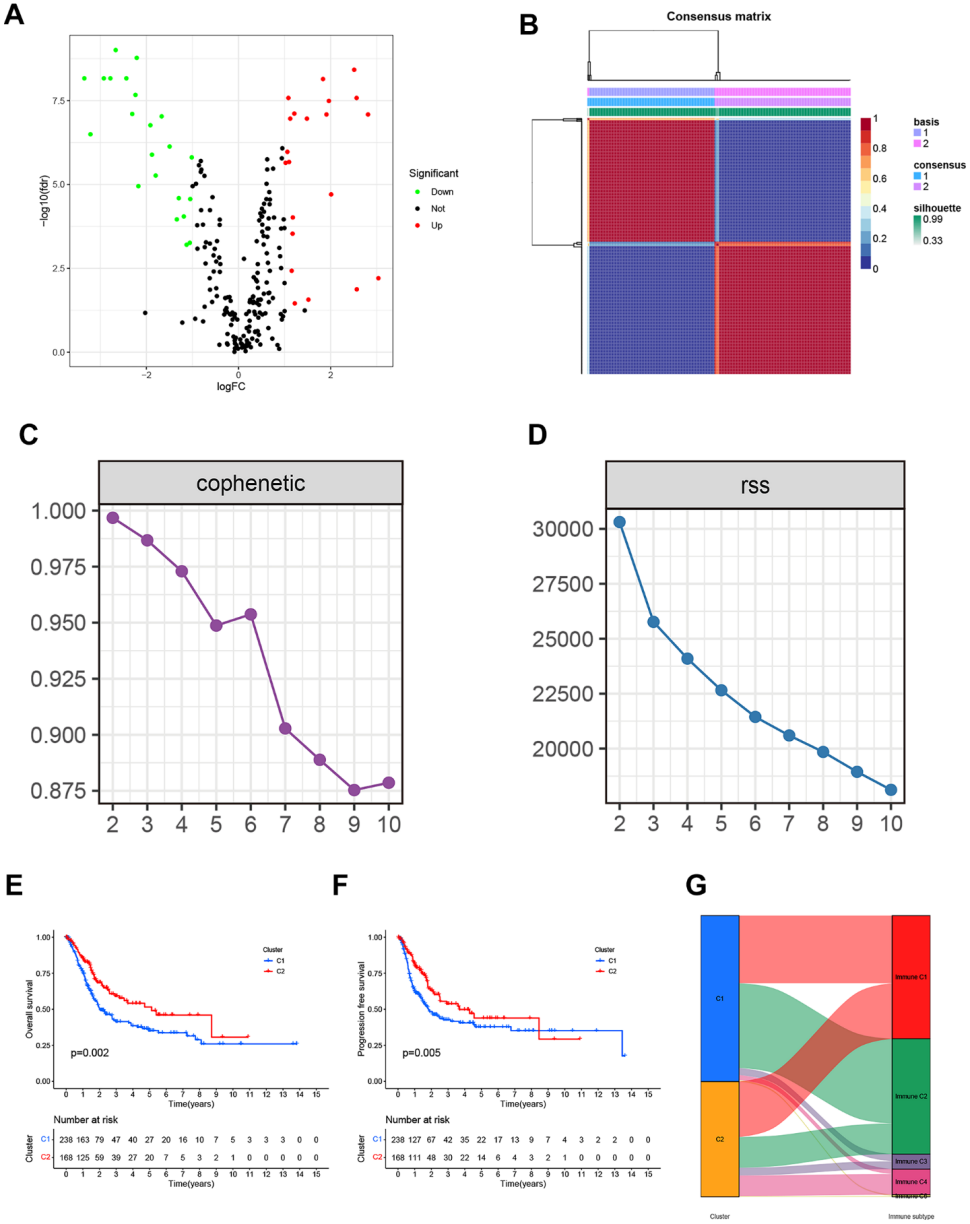
(**A**) The Volcano plot of differentially expressed genes from the BLCA patients in the TCGA database. (**B**) Non-negative matrix factorization (NMF) algorithm-based Consensus map clustered. (**C**) The stability of the cluster obtained from NMF was determined by the cophenetic correlation coefficient. (**D**) The clustering performance of the model was measured by RSS. (**E**, **F**) The statistical differences between C1 and C2 in overall survival (OS) and progression-free survival (PFS). (**G**) The mapping relationship of C1 and C2 to molecular subtypes was exhibited in the Alluvial plot.

Figure 1 displays the flowchart of analyses. Transcriptomic data was acquired from the TCGA-BLCA database, consisting of 414 BLCA samples and 19 samples of adjacent non-cancerous tissue. Finally, the transcriptome data of 406 BLCA patients were obtained after excluding cases with clinical information. Our research primarily centered around a collection of 275 genes associated with OS, where we extensively examined and compared their expression patterns. In order to guarantee the integrity and precision of the data, our initial focus was on resolving batch effects in the TCGA dataset, which was then followed by the normalization of the data. Table 1 provides a detailed account of the 43 differentially expressed genes (DEGs) that were identified between BLCA and normal bladder tissues through this procedure. Illustrated are the clinic features of BLCA individuals obtained from the TCGA and GEO databases. It is worth mentioning that most of the individuals in both groups are over the age of 65 and primarily male. Additionally, the data reveals a significant percentage of patients in advanced phases (III-IV) and with tumors of high grade, specifically within the TCGA-BLCA group. The differentially expressed genes (DEGs), which exhibited either increased or decreased expression, were visually depicted using volcano plots (as shown in Figure 2A). This graphical representation offered a straightforward and succinct visualization of their expression patterns.

**Table 1:** Clinicopathological characteristics of BLCA patients from the TCGA and GEO databases.

**Characteristics**	**TCGA-BLCA cohort N=406**	**GSE31684 N=93**
**Age**		
<=65	160 (39.41%)	28 (39.41%)
>65	246 (60.59%)	65 (60.59%)
**Gender**		
Female	107 (26.35%)	25 (26.88%)
Male	299 (73.65%)	68 (73.12%)
**Grade**		
High	383 (94.33%)	87 (93.50%)
Low	20 (4.93%)	6 (6.50%)
unknow	3 (0.74%)	0 (0.00%)
**Stage**		
I-II	131 (32.27%)	74 (79.57%)
III-IV	273 (67.24%)	11 (11.83%)
unknow	2 (0.49%)	8 (8.60%)
**T**		
T0-T2	122 (30.05%)	NA
T3-T4	251 (61.82%)	NA
unknow	33 (8.13%)	NA
**M**		
M0	195 (48.03%)	NA
M1	11 (2.71%)	NA
unknow	200(49.26%)	NA
**N**		
N0-N1	282 (69.46%)	NA
N2-N3	82 (20.2%)	NA
unknow	42 (10.34%)	NA

### NMF algorithm-based molecular subtypes of OS-related genes

**Figure 3:**
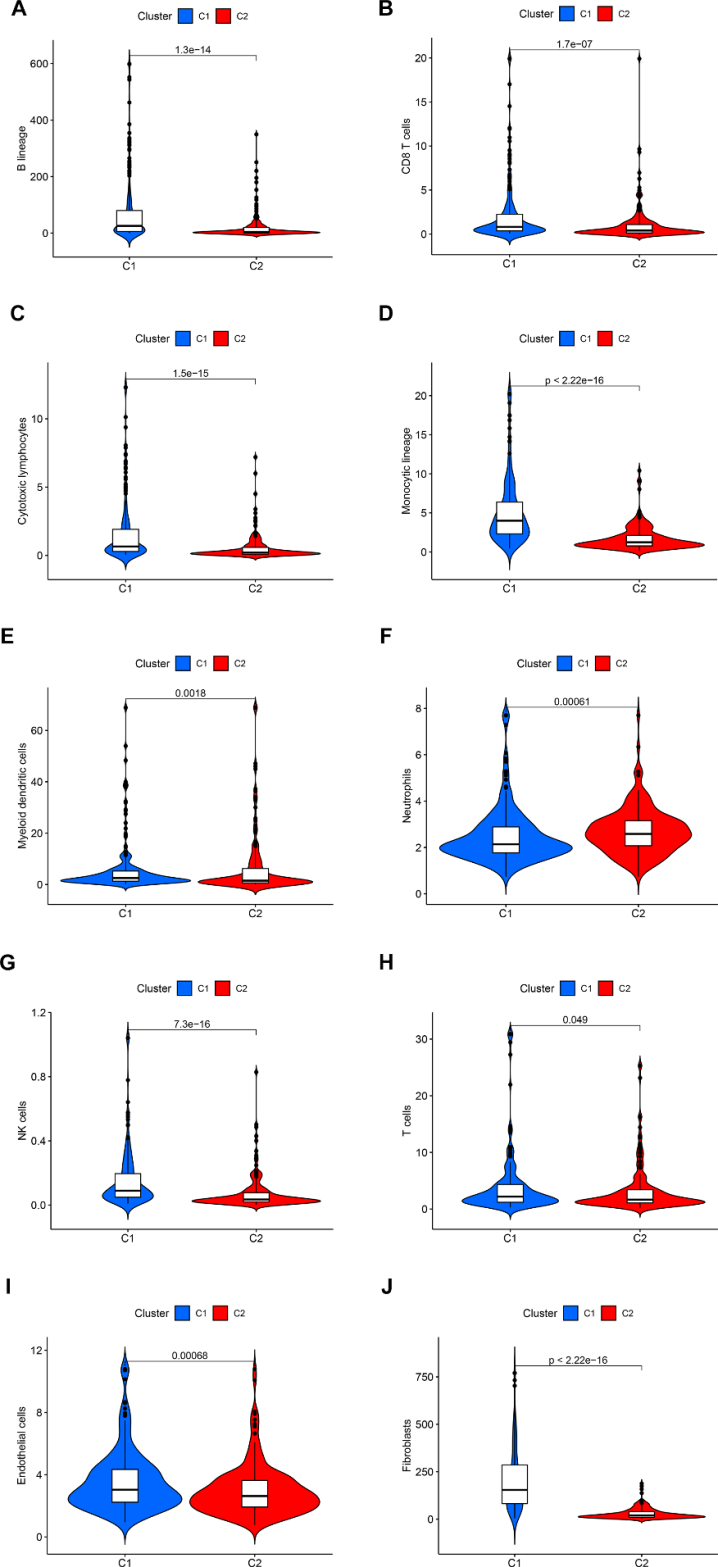
The statistical difference in the immune cells (**A**–**H**) and stromal cells (**I**, **J**) from the tumor microenvironment (TME) between C1 and C2.

By employing covariance and RSS, we identified two clusters, namely C1 and C2, where k=2 was determined to be the optimal clustering parameter considering stability and performance, as depicted in Figure 2B–2D. Figure 2E, 2F depicted that the C2 cluster exhibited enhanced overall survival (OS) and progression-free survival (PFS) in comparison to C1, as indicated by the Kaplan-Meier analysis. Furthermore, Figure 2G showcases the allocation of DEGs across the traditional immune subtypes C1-C6, encompassing wound repair, IFN-gamma predominant, inflammatory, lymphocyte-depleted, immunologically silent, and TGF-beta predominant categories. The different subcategories are associated with particular types of immune cells and indicators, which have an impact on the patient’s prognosis and how they respond to treatment. Figure 3 shows that there were noticeable differences in immune and stromal scores between C1 and C2, highlighting the grouping of OS-related genes and their connection to various immune cell clusters.

### Developing and validating a prognostic prediction model for OS-related genes

**Figure 4:**
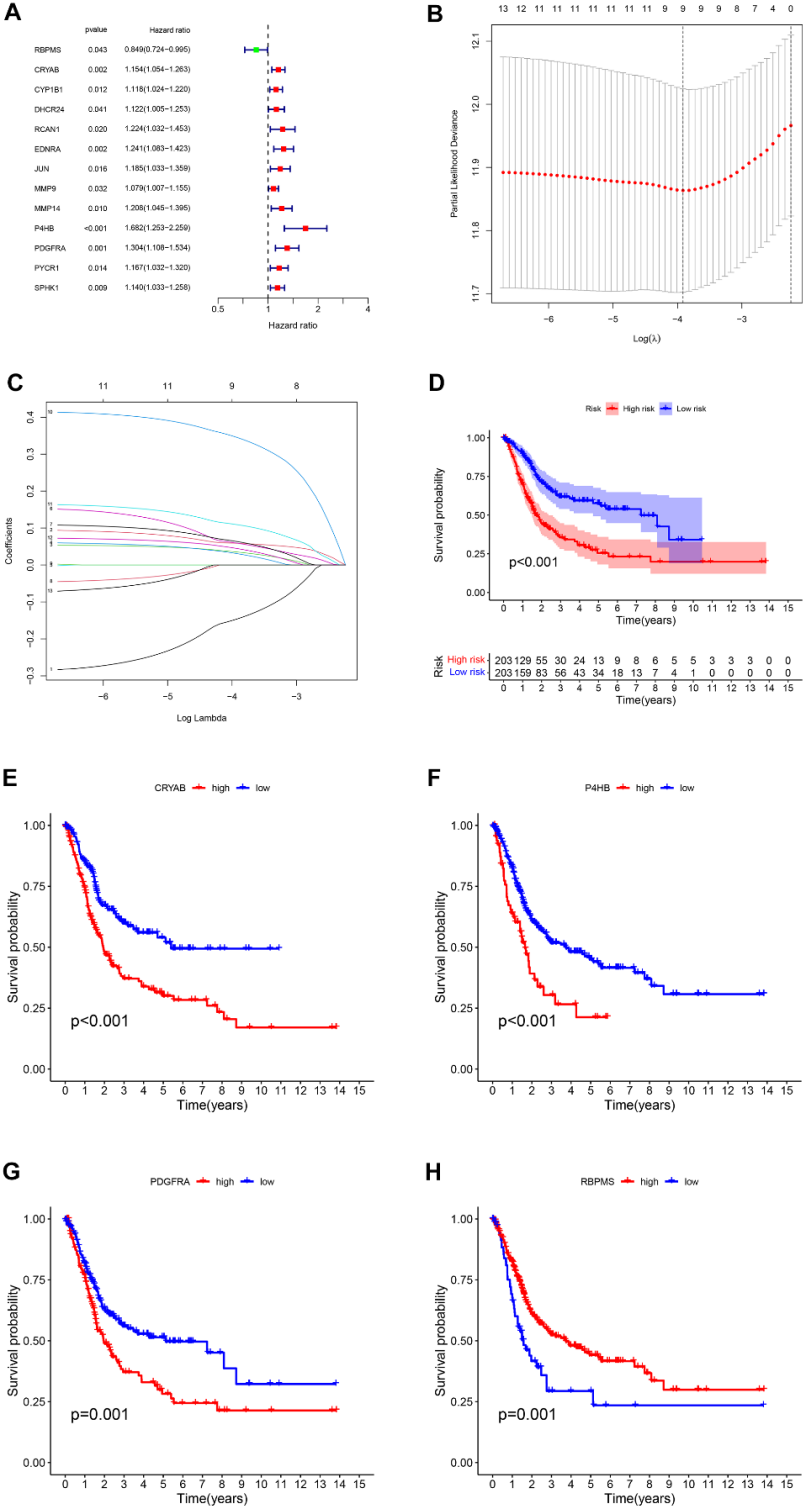
(**A**) The survival showed as Hazard ratio was determined by univariate Cox regression analysis in Forest plot. (**B**) Ten-fold cross-validation for tuning parameter selection in lasso regression. Vertical lines are drawn from the best data according to the minimum criterion and 1 standard error criterion. The vertical lines on the left represent the final 4 genes identified. (**C**) LASSO coefficient profiles of 13 OS-related genes. The 10-fold cross-validation value is marked by a vertical line. (**D**) The statistical difference exists in the overall survival between high-risk and low-risk groups as shown in the Kaplan-Meier curve. (**E**–**H**) The different overall survival between low and high CRYAB, P4HB, PDGFRA, and RBPMS expression was shown in the Kaplan-Meier curve respectively.

We conducted a univariate Cox regression analysis using 43 differentially expressed genes (DEGs) (see Supplementary Table 2 and Figure 4A). In order to avoid overfitting, we applied Lasso regression to these candidate genes (Figure 4B). Cross-validation was also used to determine the ideal value of the penalty parameter, and 13 genes associated with OS were identified (Figure 4C). Following the implementation of multivariate Cox regression, we ultimately recognized four genes (RBPMS, CRYAB, P4HB, PDGFRA) that are crucial for constructing the model. CRYAB, P4HB, and PDGFRA were regarded as unfavorable prognostic markers (HR > 1), while RBPMS was regarded as a favorable prognostic marker (HR < 1) (Supplementary Table 3).

The risk score for each sample was determined using the formula: Risk Score = -(0.2379 × RBPMS) + (0.1077 × CRYAB) + (0.4696 × P4HB) + (0.2422 × PDGFRA).

The tumor samples were categorized into high-risk and low-risk groups based on the median risk score (1.02) serving as the threshold. According to the findings from the Kapan Meier curve (Figure 4D), individuals categorized as high-risk experienced shorter overall survival durations compared to those classified as low-risk. A notable distinction was observed in overall survival (OS) between the two groups of patients based on the varying levels of expression of the four genes (Figure 4E–4H).

**Figure 5:**
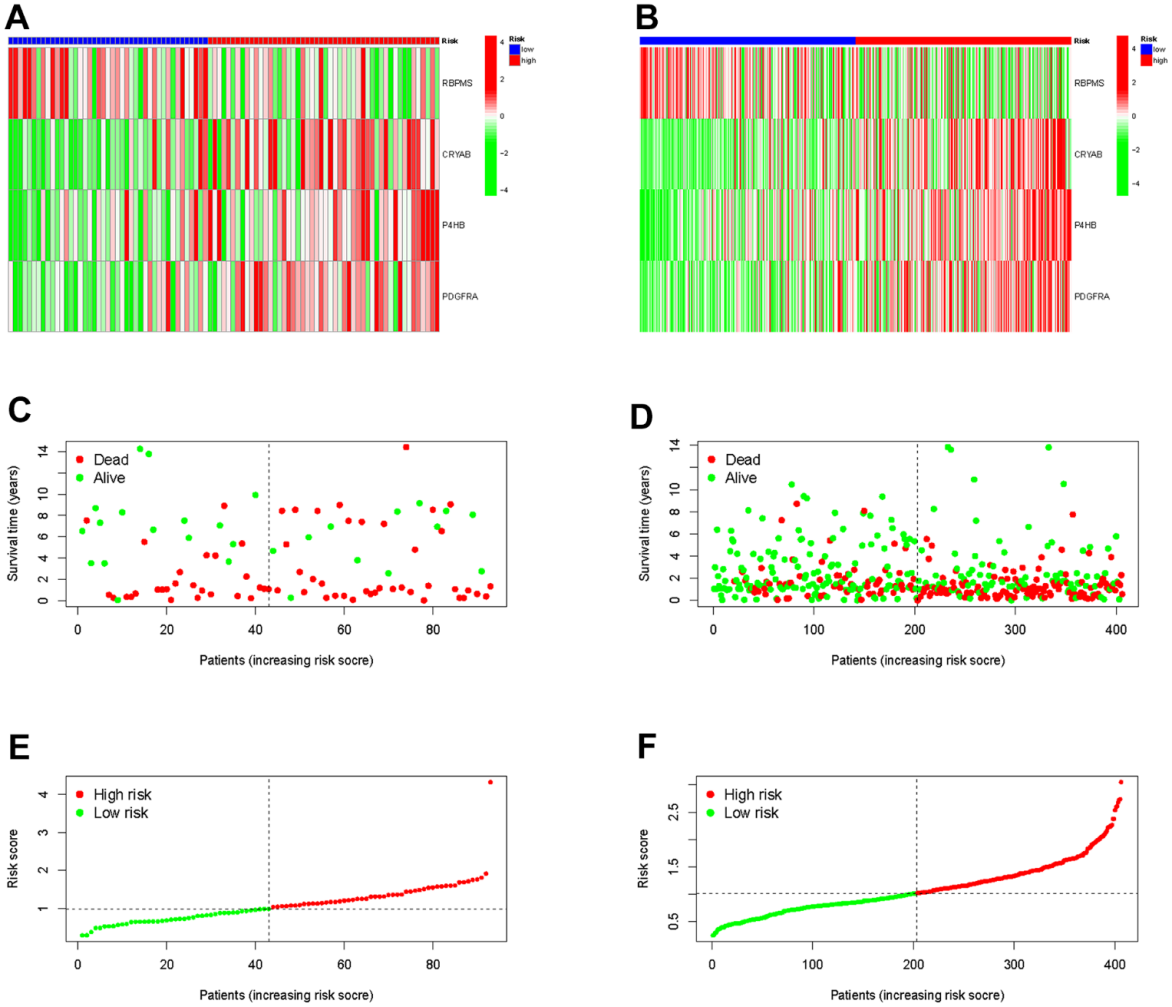
Validation the prognostic predictive value of risk scores in the TCGA cohort (**A**) and GEO cohort (**B**). Survival status and duration of BLCA patients in the TCGA cohort (**C**) and GEO cohort (**D**). The polygenic model risk score distribution in the TCGA cohort (**E**) and GEO cohort (**F**).

Furthermore, we demonstrated RS in the TCGA group (Figure 5A), the survival condition (Figure 5C), and the distribution of polygenic model RS among BLCA individuals (Figure 5E). Subsequently, we validated these findings in the external GEO group (Figure 5B, 5D, 5F).

### Construction of prognostic nomogram

**Figure 6:**
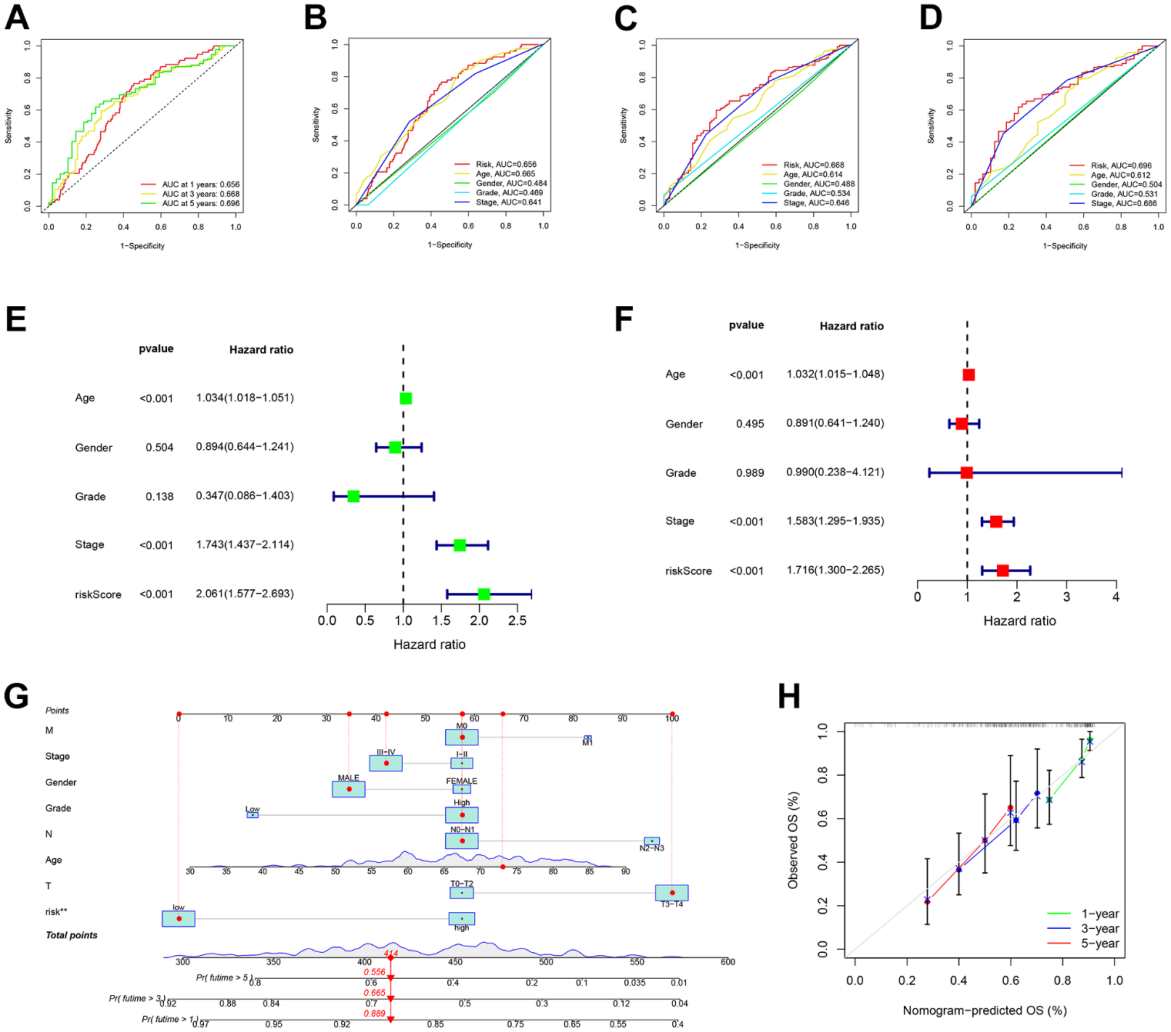
**Risk signatures prognosis efficiency validation.** (**A**) The predictive value of prognostic features was validated in the ROC analysis. The area under the curve (AUC) of the risk score to predict the overall survival at 1, 3, and 5 years and (**B**–**D**) corresponding other clinicopathological factors. (**E**) Univariate and (**F**) Multivariate Cox regression results for overall survival. (**G**) The predicted survival of BLCA patients by Nomogram, and (**H**) 1-, 3-, and 5-year nomogram calibration curves.

In Figure 6A, the analysis of the Receiver Operating Characteristic (ROC) curve for our predictive model revealed AUC values of 0.656, 0.668, and 0.696 for the 1-year, 3-year, and 5-year rates of overall survival, respectively. In order to confirm the importance of the risk score as a significant predictor among different clinicopathological features, we included variables such as sex, tumor grade, and TNM stages in the AUC analysis for 1-year (Figure 6B), 3-year (Figure 6C), and 5-year (Figure 6D) overall survival. The combined factors resulted in lower AUC values compared to the risk score alone.

In terms of clinical aspects, prognosis is influenced by factors including age, gender, stage, and grade. Significant disparities were observed in the overall survival outcomes between univariate and multivariate Cox regression, as depicted in Figure 6E, 6F. In order to assess the accuracy of the model’s predictions, we created a predictive nomogram that included the risk score and clinicopathological characteristics (Figure 6G). As an example, a male who is 73 years old and diagnosed with BLCA, classified as T3N0M0 and placed in the low-risk category, as per the model, is estimated to have survival probabilities of 89.9%, 66.5%, and 55.6% at 1, 3, and 5 years respectively. This estimation is based on a total nomogram score of 414. However, the calibration plot suggested a potential underestimation of the 3-year survival percentage (Figure 6H).

### Functional analysis of OS-related genes

**Figure 7:**
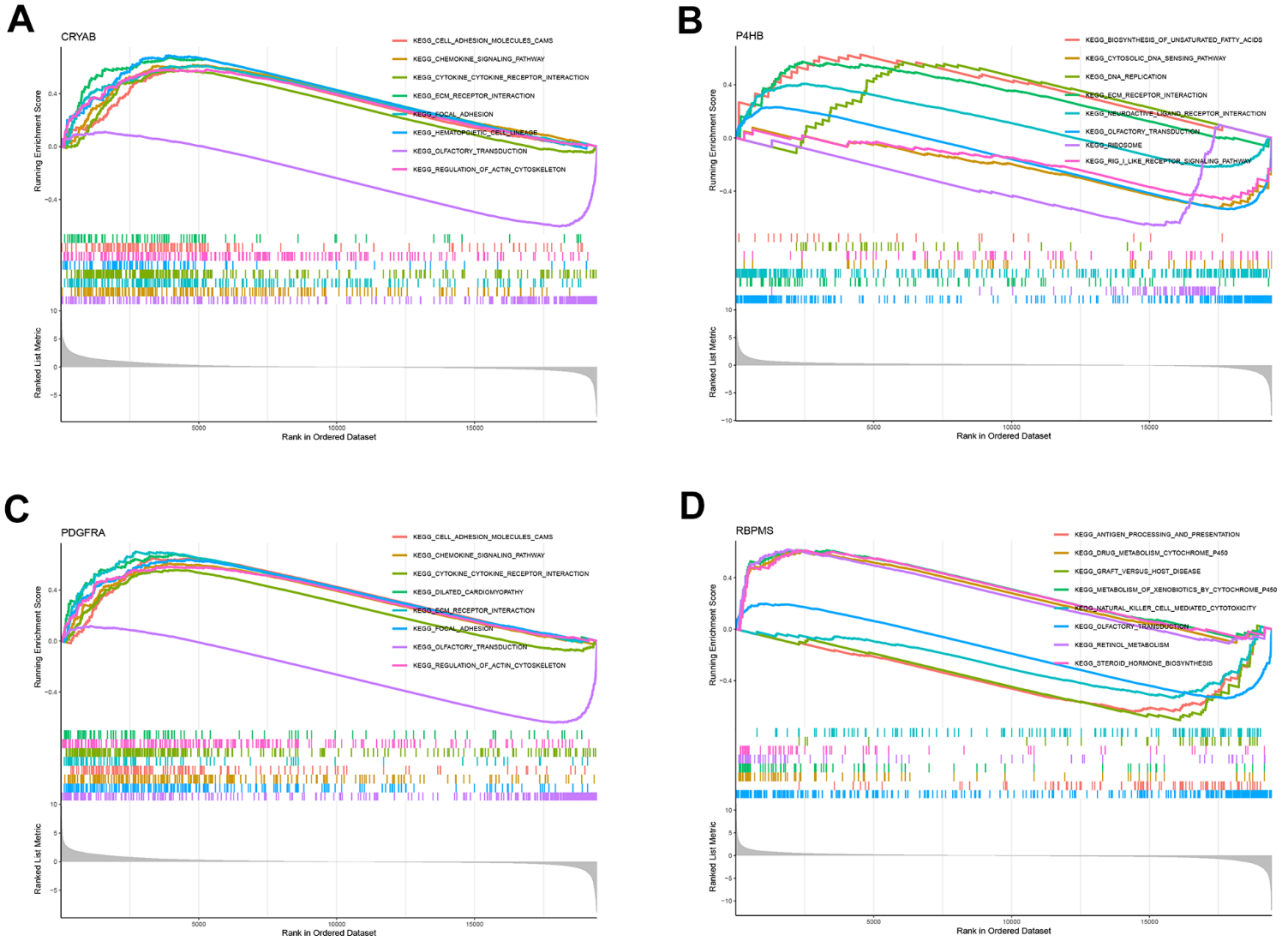
**GSEA for samples with high and low expression of 4 central genes.** (**A**) The enriched gene set obtained from KEGG for samples with high CRYAB expression. (**B**) The enriched gene set obtained from KEGG for samples with high P4HB expression. (**C**) The enriched gene set obtained from KEGG for samples with high PDGFRA expression. (**D**) The enriched gene set obtained from KEGG for samples with high RBPMS expression.

All samples were classified into low or high-expression groups based on the median expression of the hub genes. Following that, an analysis called Gene Set Enrichment Analysis (GSEA) was performed, which unveiled the presence of functional enrichment within these genes. According to the KEGG pathway analysis, an increase in CRYAB expression was linked to particular signaling pathways, particularly the interaction between cytokines and cytokine receptors, as well as the chemokine signaling pathways (Figure 7A). Likewise, heightened P4HB expression was found to be associated with the activation of the cytosolic DNA sensing pathway, along with other pathways (Figure 7B). Two distinct pathways, namely the chemokine signaling pathway and the cytokine-receptor interaction system (Figure 7C), were associated with elevated expression levels of PDGFRA. In addition, increased RBPMS expression was observed to be linked to the activation of pathways including cytochrome P450-mediated xenobiotic metabolism and retinol metabolism (Figure 7D). The results highlight the intricate interaction between the expression of these central genes and their participation in different crucial signaling pathways within the cellular environment.

### Association of risk characteristics with clinicopathological variables

**Figure 8:**
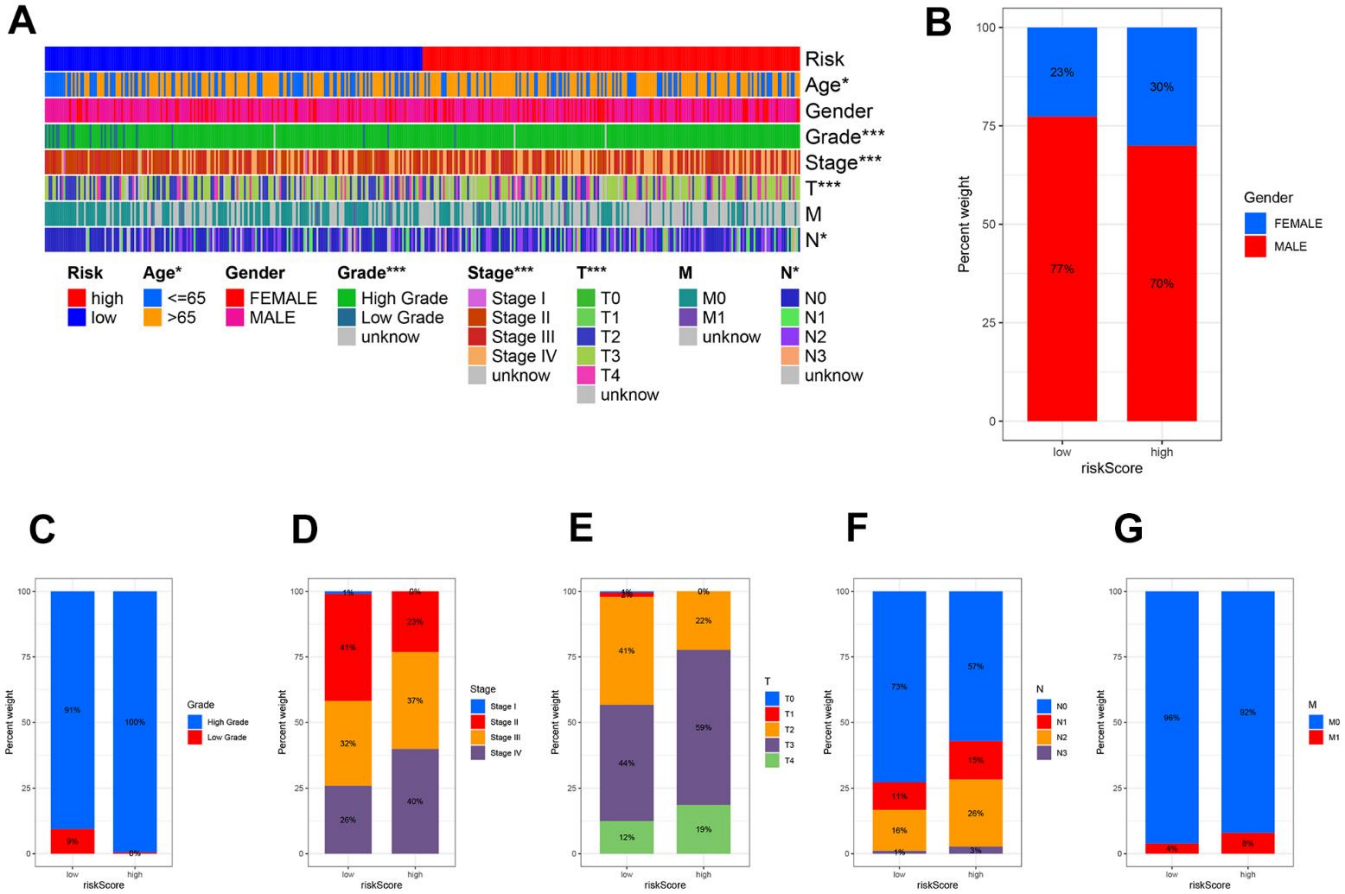
**The clinicopathologic feature of the prognostic risk characteristics.** (**A**) The clinicopathologic feature and corresponding risk scores were shown in the heatmap. The clinicopathologic feature incidence (**B**) Gender, (**C**) Tumor Grade, (**D**) Tumor stage, (**E**) Stage T, (**F**) Stage N, and (**G**) Stage M in the low and high-risk groups.

Using clinicopathological features, we created a plot (Figure 8A) to investigate the relationship between risk and clinicopathological variables. Gender (Figure 8B), tumor grade (Figure 8C), tumor stage (Figure 8D), stage T (Figure 8E), stage N (Figure 8F), and stage M (Figure 8G) exhibited variations that aligned with clinical results. The results of these findings, along with the outcomes of univariable and multivariable regression analysis, suggest that our risk score serves as a reliable prognostic indicator, regardless of other clinical factors.

### Association of risk signature with TMB

**Figure 9:**
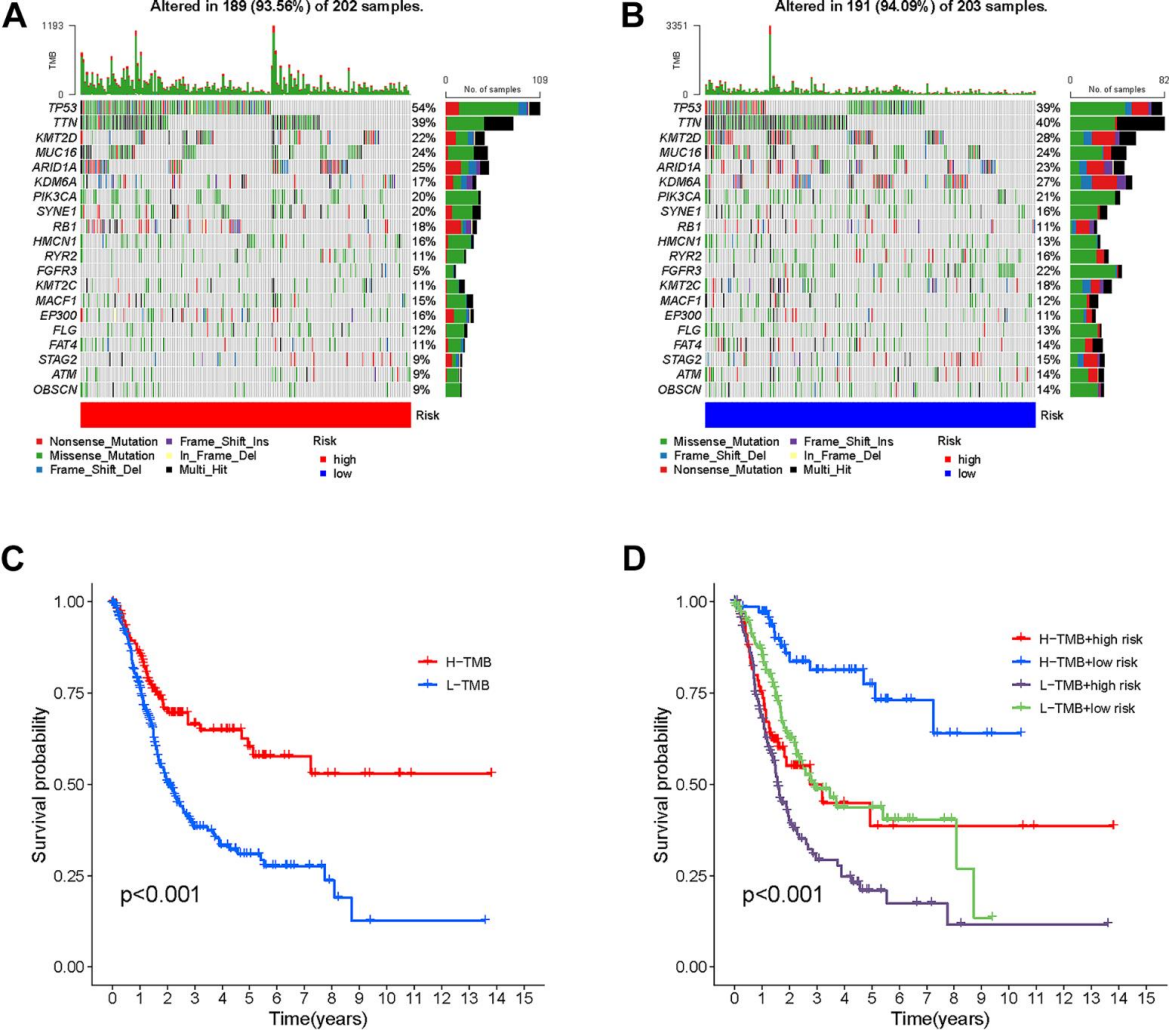
**Correlation between risk score and TMB.** High risk score. High-risk score (**A**) and low-risk score (**B**) were shown in the established oncoPrint. (**C**) High TMB and low TMB groups were shown in the Kaplan-Meier curve. (**D**) Kaplan-Meier curve stratification based on TMB and risk signature.

In order to investigate the correlation between risk score and gene mutations, our initial analysis focused on examining the prevalence of gene alterations within both high-risk and low-risk groups. Next, our attention shifted towards visualizing the waterfalls, which represent the top 20 genes exhibiting the highest occurrence of somatic mutations in each group (Figure 9A, 9B). Notably, the mutation profiles of significantly mutated genes (SMGs) indicated a higher prevalence of TP53 mutations in the high-risk group (54% as opposed to 39%), whereas FGFR3 mutations were found to be more frequent in the low-risk group (22% compared to 5%). This discrepancy is attributed to TP53’s known association with an increased cancer risk.

The results of the Kaplan-Meier analysis showed a positive correlation between elevated TMB levels and extended overall survival (OS) (Figure 9C). The TMB risk of patients was used to categorize them into four groups. The plot obtained indicated that TMB did not impact the predictive precision of the risk score (Figure 9D). The results confirm that risk scores are able to accurately forecast the influence of immunotherapy and function as a dependable indicator of prognosis.

### Risk signature in TIME context

**Figure 10:**
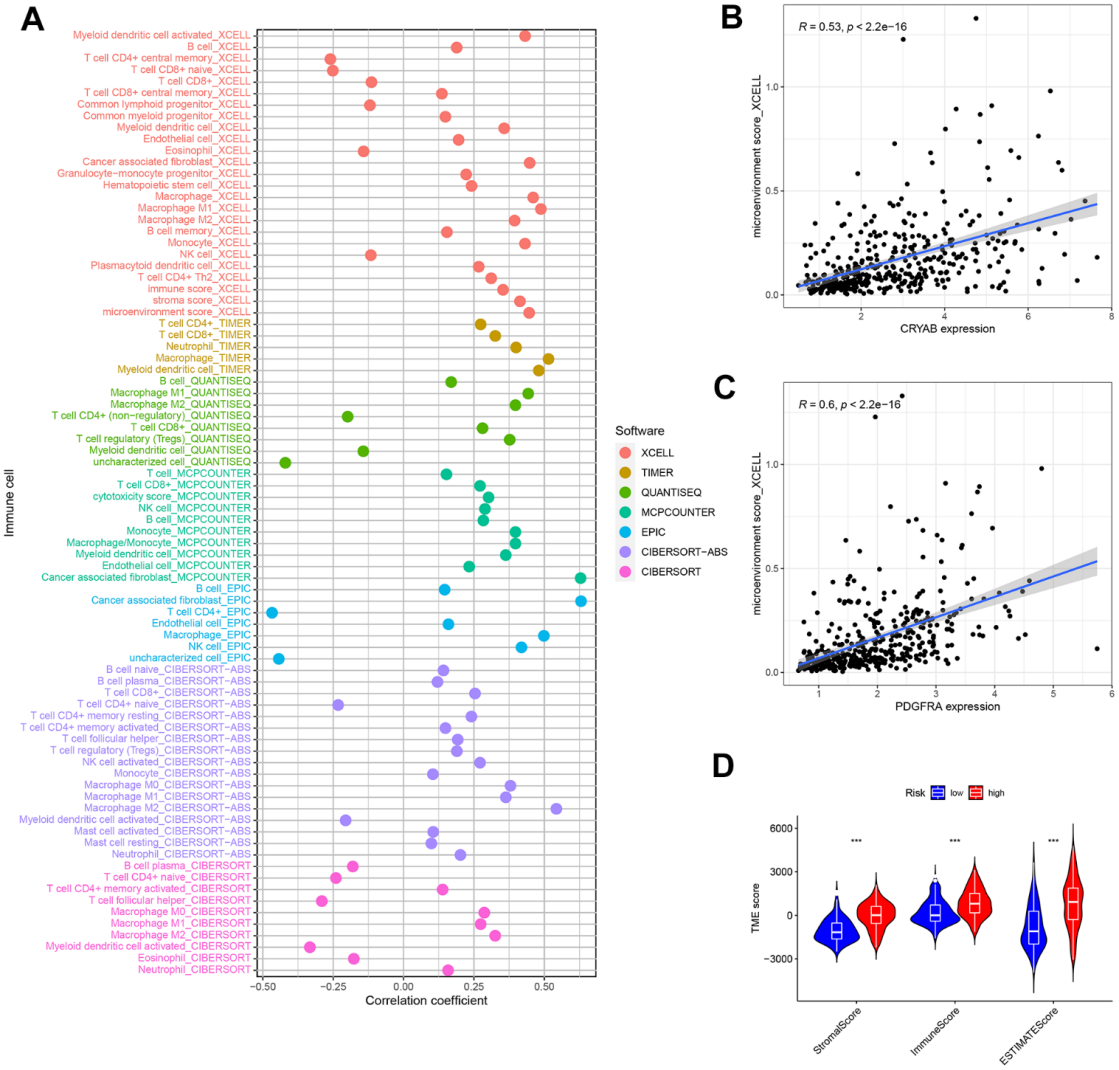
**Tumor-infiltrating cells abundance estimation.** (**A**) The significantly stronger correlation exists in the high-risk group as shown by the Spearman correlation analysis. (**B**, **C**) The correlation between the microenvironment and CRYAB and PDGFRA expression. (**D**) The TME score in the low and nigh risk groups.

In order to examine the correlation between the risk score and tumor immune microenvironment (TIME), we utilized seven distinct approaches, offering a thorough examination of the infiltration of immune cells and stromal cells in connection with the risk score (Figure 10A). The XCELL examination indicated a favorable association between the microenvironment rating and the levels of CRYAB and PDGFRA expression (Figure 10B, 10C). This suggests that the microenvironment score is directly related to the expression of these genes.

Moreover, the ESTIMATE technique demonstrated a significant rise in scores among patients at high risk. Significant variations in ESTIMATE scores, immune scores, and stromal scores were observed between the high-risk and low-risk groups, as depicted in Figure 10D. The observed pattern indicates a robust correlation between elevated risk scores and heightened participation of immune and stromal cells in the tumor microenvironment, emphasizing the potential importance of these discoveries in comprehending and controlling bladder cancer.

### Biological function and signal pathway enrichment analysis

**Figure 11:**
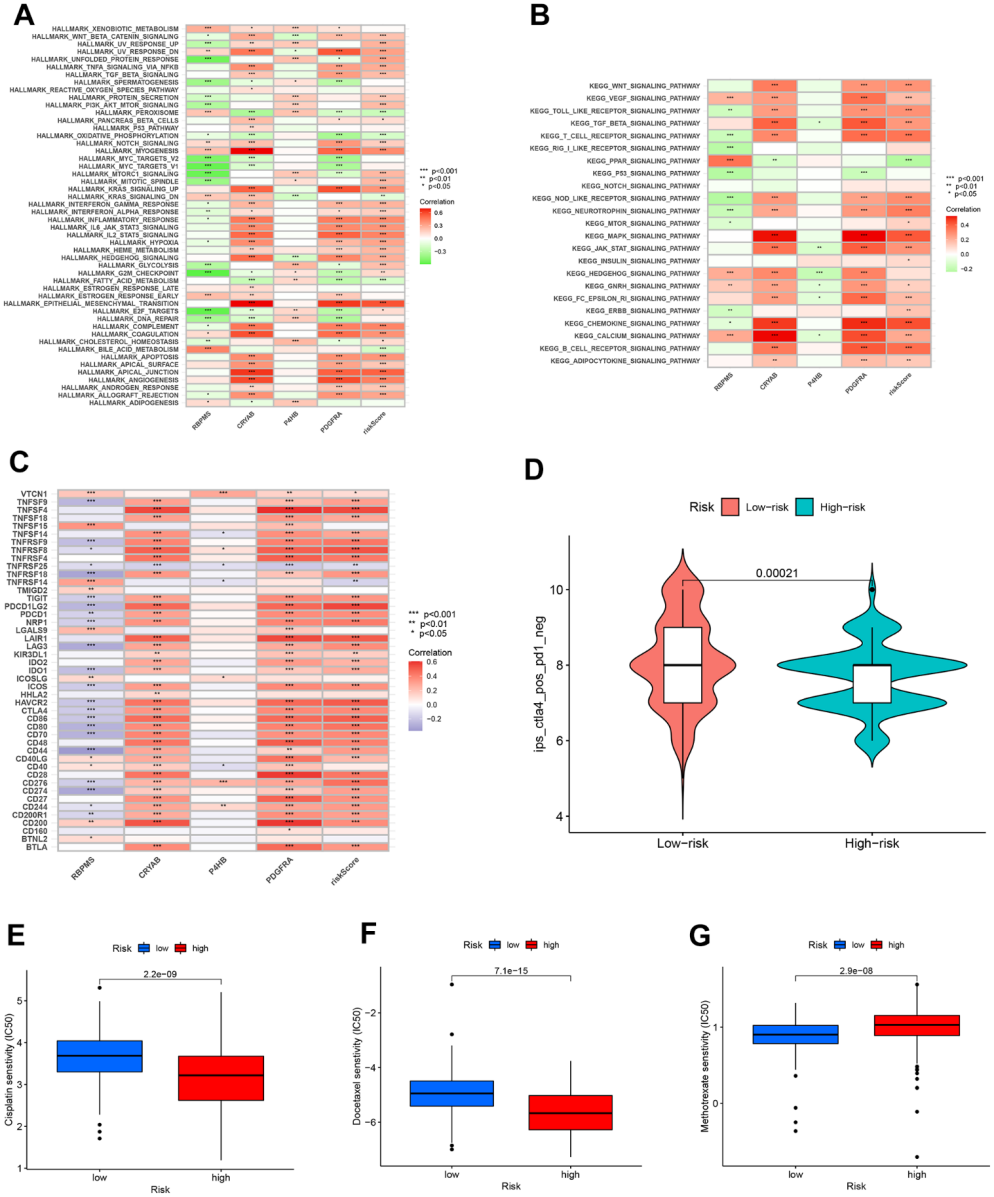
Enrichment pathways for GSVA. The correlation between pathways (**A**), immune hallmarks (**B**) and risk scores in the KEGG database. (**C**) The correlation between immune checkpoint blockade genes and risk scores to predict immunotherapy response. (**D**) The chemotherapy efficiency estimation in the IPS score distribution map. (**E**) Cisplatin, (**F**) Docelaxel and (**G**) methotrexate sensitivity in the low and high groups.

The GSVA yielded valuable information about the functional traits of the high-risk cohort. Our findings indicated that individuals in this group exhibited enhanced activity in several key biological pathways. Significantly, there was a rise in apical junction and epithelial-mesenchymal transition pathways. Furthermore, there was evidence of activation in both the MAPK pathway and the chemokine signaling pathway, as depicted in Figure 11A, 11B. The findings indicate a heightened and active tumor microenvironment in the high-risk category, marked by notable pathway activations that may impact the advancement of the disease and the response to treatment.

### Sensitivity of immunotherapy and chemotherapy prediction

Additionally, our efforts encompass forecasting the responsiveness of immunotherapy. A total of 47 genes associated with checkpoint blockade were obtained from Supplementary Table 4. Figure 11C indicated that the risk scores showed a positive correlation with several genes associated with checkpoint blockade, including TNFSF4, PDCD1LG2, LAIR1, CTLA4, and CD86. According to our predictive model, patients belonging to the CTLA4 positive and PD-1 negative groups (Figure 11D) exhibit a higher IPS score when they are classified as low-risk. This suggests that individuals with a minimal risk might be suitable candidates for receiving CTLA4 inhibitors.

Furthermore, we assessed the effectiveness of BLCA when used alongside conventional chemotherapy medications like cisplatin, docetaxel, and methotrexate. Figure 11E, 11F demonstrated a notable decrease in IC50s among high-risk patients for both Cisplatin and Docelaxel. This suggests that individuals with a greater susceptibility are more receptive to the chemotherapy medications paclitaxel and cisplatin compared to those with a lower susceptibility. Figure 11G shows that patients classified as low-risk had a more favorable response to methotrexate therapy compared to those classified as high-risk. This suggests that patients with a lower risk profile exhibit a greater response to methotrexate compared to patients with a higher risk profile.

## DISCUSSION

The present investigation evaluated 275 genes associated with OS in the 359 TCGA BLCA samples. To separate the two subclusters, the NMF method, an innovative clustering technique, was employed. We discovered that there are noticeable variations in diverse tumor-infiltrating cells and supportive cells in the tumor microenvironment between C1 and C2. Patients with distinct subclusters exhibit significant differences in both OS and PFS compared to the remaining group. After conducting a series of regression analyses, the prognostic prediction model was built using RBPMS (RNA Binding Protein with Multiple Splicing), CRYAB (Crystallin Alpha B), P4HB (Prolyl 4-Hydroxylase Subunit Beta), and PDGFRA (Platelet Derived Growth Factor Receptor Alpha) as the fundamental components.

According to reports, RBPMS plays a role in controlling RNA splicing, transportation, positioning, and durability. According to Rastgoo [23], the connection between RBPMS and miR-138 played a role in the drug sensitivity of multiple myeloma (MM) with the influence of the enhancer of zeste homolog (EZH2). Additionally, the reintroduction of RBPMS resulted in the resensitization of previously resistant MM cells. CRYAB binds with p53 to prevent its movement to the mitochondria, consequently indirectly restraining its pro-apoptotic impact on the apoptotic Bcl-2 molecule. In addition, CRYAB was discovered to hinder p53-triggered cell death, which is facilitated by the calcium-stimulated Raf/MEK/ERK signaling pathway, through the inhibition of Ras activation. Additionally, it has the ability to hinder UVA cell death by engaging in the control of PKCα1pha and Raf/MEK/ERK signaling pathway proteins [24]. Certain types of cancer have been found to exhibit an increase in P4HB, and an excessive expression of P4HB could potentially accelerate the advancement of malignant tumors, such as gastric cancer, clear cell renal cell carcinoma, and colon cancer. The endoplasmic reticulum stress response pathways have been linked to increased sensitivity to chemotherapy in glioblastoma multiforme through the inhibition of P4HB [25]. Mutations in PDGFRA [26] result in the development of various types of gastrointestinal mesenchymal tumors with diverse characteristics. Previously known as intestinal neurofibromatosis/neurofibromatosis 3b (INF/NF3b), this disease is now classified as a familial gastrointestinal stromal tumor (GIST) due to its genetic makeup, which was identified when GIST was the sole PDGFRA-mutant tumor found in the gastrointestinal tract. PDGFRA mutations were also detected in inflammatory fibroid polyps. Later on, there were reports of gastrointestinal CD34+ ‘fibrous tumors’ with an uncertain classification in the presence of a germline PDGFRA mutation. These findings indicate a robust correlation between the gene and the formation of certain tumors.

Prior to the initiation of cancer treatment, certain research has indicated that cancer patients may exhibit a diminished level of antioxidants and an elevated degree of oxidative stress [27]. Oxidative stress state has prognostic relevance to cancer, and may significantly help to prescribe appropriate treatment schemes for patients. The excessive release of ROS in the persistent inflammatory cells will attract additional activated immune cells, resulting in the escalation of the imbalance process and ultimately causing the formation of precancerous lesions. When cells produce enough ROS to overpower internal antioxidant reactions, it can cause irreversible oxidative harm to nucleic acids, lipids, and proteins. This damage can result in genetic and/or epigenetic alterations, ultimately causing an imbalance in oncogenes and tumor suppressor genes [28]. Several researches indicate that the decrease in NOX1 expression in urothelial carcinoma results in a decline in the generation of reactive oxygen species (ROS) and a resistance to programmed cell death, ultimately contributing to the progression of bladder cancer [29]. Furthermore, ROS plays a vital role in the regulation of angiogenesis. Endothelial cell proliferation is facilitated by NOX4-induced production of H2O2, while NOX2 inhibits apoptosis and enhances endothelial cell survival. Moreover, ROS has been linked to the phosphorylation of the cadherin/catenin cell-cell adhesion complex through vascular endothelial growth factor (VEGF). ROS-induced phosphorylation of cadherin/catenin causes the dismantling of endothelial cells, thereby promoting their migration [30]. Photodynamic therapy targeting oxidative stress has been used to treat urothelial carcinoma and achieved clinical benefits. In a small group of 17 patients with recurrent non-muscle invasive bladder cancer, photodynamic therapy using hexaminolevulinate was evaluated in a clinical trial. 21 months later, 12% of the patients were found to be free of tumors [31]. The investigation of TLD1433 has concluded its phase I study, and currently, a more extensive phase II trial is in progress in the BCG-unresponsive context (NCT03945162).

Numerous genes related to oxidative stress in the model have been documented to be linked to the onset and progression of urethral epithelial carcinoma. The upregulation of circRBPMS hinders the miR-330-3p/retinoic acid-induced 2 axis, thereby suppressing the growth and spread of bladder cancer cells [32]. CRYAB overexpression leads to a decrease in the epithelial-mesenchymal transition, thereby inhibiting the migration and invasion of the T24 and J82 BC cell lines [33]. P4HB inhibitor coordinated Gemcitabine exerted an anticancer effect by raising cellular ROS content and promoting cell death [34]. Urothelial cancer exhibits a robust PDGFRA immunohistochemical signal and BLCA with KIT and PDGFRA expression, demonstrating varied differentiation into various morphological types [35].

To enhance the applicability of the model in clinical practice, a nomogram and prediction of drug sensitivity were executed. Pierre Denoix proposed Tumor-Node-Metastasis as the preferred method for predicting the outcome of solid tumors [36], which is widely considered the benchmark. Using the existing gathered samples, we developed a nomogram by combining prognostic factors and medical variables. TMB is the abbreviation for the overall count of genetic alterations per million bases in the coding section of the gene under examination in the genome of tumor cells. It was first used as a biomarker to assess the effectiveness of Ipilimumab or Tremelimumab treatments in patients with advanced melanoma. In clinical practice, individuals with elevated TMB expression experience greater advantages from the use of immune checkpoint inhibitors. As anticipated, patients with elevated TMB and high TMB/low risk, who are categorized based on OS-associated genes, exhibit a more favorable prognosis. Chemotherapy serves as the primary supplementary therapy for bladder urothelial carcinoma, while immune checkpoint inhibitors emerge as the most encouraging novel treatment. Significant correlations were observed between the risk scores and the immune checkpoints PD-L1/CD274, CTLA4, and B7-H3/CD276. According to the National Comprehensive Cancer Network’s ‘Clinical Practice Guidelines in Oncology’ for bladder cancer, the use of PD-1/PD-L1 blocking antibodies such as Atezolizumab [37, 38], Nivolumab [39], Avelumab [40], and Pembrolizumab [41, 42] is recommended from a clinical perspective. The ongoing progress involves the utilization of tremelimumab, durvalumab, and CTLA4 antibody for treating metastatic bladder cancer [43]. The clinical approval of the anti-B7-H3 mAb antibody chemically linked with the anti-CD3 antibody has been confirmed [44]. Several investigations have been conducted on the use of PD-1 in conjunction with CTLA-4 for cancer therapy; however, the outcomes have been unsatisfactory according to studies [45, 46]. The correlation analyses and distribution of IPS indicated that the risk score is a useful predictor for the responsiveness of immune checkpoint inhibitors. Specifically, samples belonging to the low-risk group exhibited a higher likelihood of positive response to CTLA4 inhibitors. Ipilimumab, a CTLA-4 antibody, may be considered for patients diagnosed with operable high-risk urothelial carcinoma who are ineligible for cisplatin treatment and have a low calculated risk score according to this model. Paclitaxel, methotrexate, and cisplatin are commonly used in chemotherapy. Paclitaxel and cisplatin exhibited higher sensitivity in the high-risk group samples, while methotrexate showed greater sensitivity in the low-risk group samples. By utilizing the predictive model, clinicians can enhance their ability to choose adjuvant treatment schemes that are better suited and more impactful. Additionally, the findings indicate that the responsiveness of bladder cancer cells to chemotherapeutic drugs can be influenced by genes associated with oxidative stress.

We applied the gene enrichment analysis to sort selected genes by functional characteristics. Several inhibitors targeting MAPK were found to have varying effects on the cell cycle during the proliferation of bladder cancer cells [47]. Moreover, p38 MAPK regulated the mRNA levels of MMP-2/9 by influencing both transcript stability and MMP-2/9 activity, which in turn affected the invasive capacity of bladder cancer [48]. In GSVA, the low-risk OS-associated group showed activation of the MAPK signaling pathway, while the HRG was linked to the WNT, VEGT, toll-like receptor, and TGF-BETA signaling pathways. When the Wnt/-catenin signaling is blocked, the growth, movement, and infiltration of the cancer cell lines Cal29 and T24 are significantly reduced [49]. Salinomycin, a potential cancer-fighting medication, triggered cell death by generating reactive oxygen species (ROS), and this process was associated with ROS-driven autophagy by controlling the PI3K/Akt/mTOR and ERK/p38 MAPK signaling pathways. Through the ERK/p38MAPK signal pathway, Toyocamycin has the ability to increase apoptosis in PC-3 cells by means of ROS mediation [29].

Despite the exploration of biomarkers related to OS in bladder urothelial carcinoma [50] in the study, our model has been extensively validated using multiple methods, ensuring its stability and effectiveness. On the other hand, we made predictions about the effectiveness of frequently utilized medications in the medical setting by considering the IC50 measurement. Additionally, we examined the relationship between the model and IPS scores and TMB, thereby enhancing its relevance to clinical decision-making. For non-negative matrix data such as mRNA transcriptome, the NMF classification method is undoubtedly more robust. GSVA analysis has the ability to identify slight variations in the manifestation of gene sets, surpassing the capabilities of GSEA analysis on its own [51]. Overall, the prognostic model offers novel perspectives for the precise detection of bladder cancer, aiding in the direction of clinical management and prediction of prognosis.

Our study also has some limitations. In order to enhance the credibility of our findings, we plan to carry out a future prospective cohort investigation, assess the expression levels, and perform functional experiments on prototype genes within tumor tissues and cells.

## CONCLUSIONS

Our study introduces an oxidative stress-based prognostic model for bladder cancer, offering insights into personalized therapy. Validated by comprehensive datasets, it identifies potential targets and advances molecular understanding, with future studies poised to enhance its clinical utility.

## Supplementary Material

Supplementary Table 1

Supplementary Tables 2-4
