# Supplementary material for: Prognostic model development and molecular subtypes identification in bladder urothelial cancer by oxidative stress signatures
Source: Aging (Albany NY). 2024 Feb 1;16(3):2591–616. doi: 10.18632/aging.205499 (PMC10911378; doi:10.18632/aging.205499)
Supplement: Supplementary Tables 2-4 [file aging-16-205499-s002.pdf]

Supplementary Table 2. 43 DEGs between BLCA tissue and normal bladder tissue.

gene	conMean	treatMean	logFC	p-Value	fdr
KLF2	50.10274	7.890132304	-2.666768106	0.0000000000046	0.000000000981
FBLN5	20.98978011	4.451141592	-2.237439754	0.000000000901	0.0000000213
PRDX4	22.83731895	46.37577408	1.021978071	0.000000329	0.00000226
RBPMS	23.889833	8.473346786	-1.495392949	0.0000000761	0.000000737
ROMO1	62.67109684	130.7096429	1.06049343	0.00000012	0.00000106
CRYAB	72.97536368	7.168302227	-3.347706126	0.000000000169	0.00000000683
CYP1B1	12.02484474	4.893396762	-1.297110098	0.00000553	0.0000256
DHCR24	34.91999705	78.73507403	1.172953018	0.000104447	0.000292726
RCAN1	35.28852932	7.128922282	-2.307443408	0.00000000518	0.0000000789
ECT2	2.514601511	9.443768588	1.909032896	0.00000000614	0.0000000817
EDNRA	9.713468684	2.792658356	-1.798347485	0.000000941	0.00000541
STX2	5.054701632	2.429435567	-1.057004766	0.000210841	0.000547672
EZH2	1.927299325	7.508680556	1.961978765	0.00000000181	0.0000000322
FANCD2	1.128269168	4.02331246	1.834272497	0.000000000269	0.00000000717
KDM6B	19.85784137	9.092895139	-1.126897184	0.000249728	0.000625789
ATP13A2	6.953754263	15.13878007	1.122384963	0.00000000937	0.000000109
FOS	917.6178958	120.9006801	-2.924071168	0.000000000224	0.00000000683
FYN	9.536013474	4.186484084	-1.187647134	0.0000245	0.0000899
ERO1A	10.16251311	21.73203189	1.096565857	0.000000301	0.00000213
HBA2	16.76924574	8.113364272	-1.047445631	0.00000599	0.0000271
HBB	39.56194094	15.63557606	-1.339280835	0.0000327	0.000109748
HMOX1	13.43978716	31.32138682	1.220637802	0.022897394	0.035087373
IL6	48.17630021	5.190759409	-3.214306077	0.0000000314	0.000000318
AQP1	110.1433847	23.81756797	-2.209284838	0.0000000000157	0.00000000167
JUN	257.5879074	68.35443722	-1.913957968	0.0000000161	0.000000171
MICB	0.894485168	3.604507875	2.010672835	0.00000408	0.0000197
MMP9	6.212235507	50.95762888	3.03611372	0.00315757	0.00622743
MMP14	58.47144684	132.302982	1.178041384	0.0000271	0.0000961
NUDT1	6.260414	14.49558679	1.211283766	0.00000000468	0.0000000768
NR4A2	32.54010779	4.732509379	-2.78154176	0.000000000169	0.00000000683
P4HB	110.3673558	233.7270467	1.082511173	0.00000000136	0.0000000263
PCNA	42.73508316	119.6077893	1.484818512	0.00000000969	0.000000109
PDGFRA	10.10898036	2.747585951	-1.879400969	0.000000152	0.00000129
PYCR1	3.055359437	21.45174211	2.811680799	0.00000000587	0.0000000817
S100A7	46.7743548	277.2080071	2.567179272	0.007214117	0.013361799
SELENOP	13.76300511	6.808018636	-1.015488621	0.00000019	0.00000155
SLC8A1	2.551852	0.565574558	-2.173755532	0.00000217	0.0000112
SOD3	52.95633947	16.65484697	-1.668861303	0.00000000742	0.000000093
ACOX2	3.002071553	0.555053517	-2.43525958	0.000000000206	0.00000000683
SLC4A11	2.914581168	6.486980035	1.154258419	0.001770036	0.003732849
SPHK1	3.010551337	8.616760846	1.517117924	0.016445166	0.027153646
CDK1	2.775738716	15.81162291	2.510041778	0.0000000000534	0.00000000379
MELK	1.797052318	10.61949296	2.563010568	0.00000000128	0.0000000263

Supplementary Table 3. Model genes for constructing prognosis model.

id	coef
RBPMS	-0.23793397221544
CRYAB	0.10767923775273
P4HB	0.469604158778852
PDGFRA	0.242248748329676

Supplementary Table 4. 47 genes related to checkpoint blockade.

IDO1
LAG3
CTLA4
TNFRSF9
ICOS
CD80
PDCD1LG2
TIGIT
CD70
TNFSF9
ICOSLG
KIR3DL1
CD86
PDCD1
LAIR1
TNFRSF8
TNFSF15
TNFRSF14
IDO2
CD276
CD40
TNFRSF4
TNFSF14
HHLA2
CD244
CD274
HAVCR2
CD27
BTLA
LGALS9
TMIGD2
CD28
CD48
TNFRSF25
CD40LG
ADORA2A
VTCN1
CD160
CD44
TNFSF18
TNFRSF18
BTNL2
C10orf54
CD200R1
TNFSF4
CD200
NRP1
